# Network Class Superposition Analyses

**DOI:** 10.1371/journal.pone.0059046

**Published:** 2013-04-02

**Authors:** Carl A. B. Pearson, Chen Zeng, Rahul Simha

**Affiliations:** 1 Emerging Pathogens Institute, University of Florida, Gainesville, Florida, United States of America; 2 Physics, The George Washington University, Washington, DC, United States of America; 3 Physics, Huazhong University of Science and Technology, Wuhan, China; 4 Computer Science, The George Washington University, Washington, DC, United States of America; Universitat Politecnica de Catalunya, Spain

## Abstract

Networks are often used to understand a whole system by modeling the interactions among its pieces. Examples include biomolecules in a cell interacting to provide some primary function, or species in an environment forming a stable community. However, these interactions are often unknown; instead, the pieces' dynamic states are known, and network structure must be inferred. Because observed function may be explained by many different networks (*e.g.*, 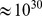 for the yeast cell cycle process [1]), considering dynamics beyond this primary function means picking a single network or suitable sample: measuring over all networks exhibiting the primary function is computationally infeasible. We circumvent that obstacle by calculating the network class ensemble. We represent the ensemble by a stochastic matrix 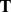, which is a transition-by-transition superposition of the system dynamics for each member of the class. We present concrete results for 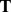 derived from Boolean time series dynamics on networks obeying the Strong Inhibition rule, by applying 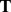 to several traditional questions about network dynamics. We show that the distribution of the number of point attractors can be accurately estimated with 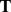. We show how to generate Derrida plots based on 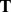. We show that 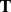-based Shannon entropy outperforms other methods at selecting experiments to further narrow the network structure. We also outline an experimental test of predictions based on 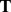. We motivate all of these results in terms of a popular molecular biology Boolean network model for the yeast cell cycle, but the methods and analyses we introduce are general. We conclude with open questions for 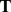, for example, application to other models, computational considerations when scaling up to larger systems, and other potential analyses.

## Introduction

Researchers across several disciplines have increasingly focused on network-based descriptions of complex behavior, with notable success in systems biology phenomena. That focus is essential to connecting knowledge across various scales – from cell chemistry to individual organisms, individuals to populations, and populations to ecosystems – and is driven by increasing availability of high-fidelity data, computational processing power to digest and test hypotheses against those data, and high-profile applications from designer pharmaceuticals to environmental policy [2]–[6]. We motivate this work and our discussion generally at the cell chemistry scale – and specifically with the yeast cell cycle process – but the methods and analyses apply at any scale, not just the microscopic.

In molecular biology, Kauffman provided one of the earliest applications of network-based thinking several decades ago [7], addressing the emergence of order in biological systems. Framing observations of organisms and their mechanics in evolutionary terms is a persistent paradigm in systems biology and an area of enduring interest (see the thousands of citations of Kauffman's omnibus work *The Origins of Order*). The last decade in particular has seen an explosion in network-oriented research in this area. Kauffman's initial explanation was structural - add enough components and interactions, and results approximating simple biological reactions reliably emerge. Researchers framed much of the subsequent work in this light, delving into particular recurring structures (“motifs” addressed in *e.g.* [8] to more recent publications like [9]), the details of how the components are added [10], the dependence of other life-like features on network properties (*e.g.*, error and attack tolerance [11]), and so on. This body of work explains *function* – what the system is observed to *do* – from the network and its properties; we call this the *network perspective*.

Work in the network perspective does not usually focus on the exact details of the dynamics associated with a particular network. Consider the work on Boolean activation-inhibition network models, of the type initially introduced for the fruit fly (*D. melangastor*) [12]. Most work on the popular research model the cell cycle of the yeast (*S. cerevisiae* – proposed in [13], and later shown to be suitably modeled by the Strong Inhibition rule [14]) has focused on stability and analogous measures (robustness, reliability, *etc.*). This work, and work on other model systems, typically starts with showing a network replicates some primary function. Other dynamic properties are then considered a consequence of that network.

What if, instead of assuming the network, one assumes that primary function? In molecular biology, this roughly means starting from a gene expression time series (microarray time course data) and asking the question: what can we say about the possible interactions that would exhibit these dynamics? That is the question we have been asking in our recent work, and we use it to frame a complementary *functional perspective*. As we have shown, it is possible to reproduce primary structural results [15]. However, when only specifying the primary function, many of the network interactions are partially constrained or unconstrained. The class of networks that support a particular primary function (also known as the neutral network [16]) can be quite large, so large as to be computational intractable when considering measures that require a calculation for each network in the class. To address this problem, we developed the measure 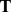 which captures, in a computationally feasible way via a single stochastic matrix, the superposition of the dynamics of each network in the class. Our results cover applying 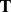 to several questions, some of which are analogous to questions asked in the network perspective and some of which are new.

First, we use 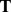 to estimate the distribution of point attractors, which has been a traditional focus of network perturbation studies. Point attractors can be used as a surrogate for overall function in an evolutionary context [17], and return to a particular attractor after some state perturbation is a common measure of network robustness (*e.g.* [18]), consistent with the loose definition of network robustness to mean minimal change in some feature under perturbations [19], [20]. The distribution and biological relevance of attractors under different models remains an area of active interest [21]–[23].

Second, we show how to approximate the Derrida plot, a popular measure of ordered versus chaotic behavior. We also apply this to the putative yeast cycle network to compare it with the 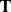 derived from the primary yeast cell cycle process. Several recent papers by Kauffman *et al.* (starting with [24] and [25]) have applied Derrida plots to the question of canalyzing update rules and other network features. We show that 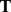-based plots can capture a function independent of an underlying network, though we note open questions about system-to-system comparisons using this measure.

Then, we quantify which experiments will likely best identify a unique network structure that supports some observed phenomena. In molecular biology, even with the downward trends in experimental cost and data analysis, the demand for data over a plethora of systems remains voracious enough that optimizing the choice of tests seems imminently practical, and at other scales – for example, ecological – extensive testing remains implausible. Other groups have used a similar Shannon entropy based approach [26], and continue to provide tools on that basis [27]. That work assumes some network constraints (minimal interactions) and targets knockout experiments; we do not assume that network constraint, and focus on initial condition experiments (though knockout experiments can also be selected).

Finally, we propose how 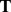 might be used for aggregate populations by making phenotypic diversity predictions, and calculating relative risk and odds ratios for particular dynamic transitions. We are not familiar with experimental work comparing the variability of natural systems and model networks for particular functions, biological or otherwise, but there is ongoing discussion about the balance of phenotypic and genotypic variation on evolutionary time scales (*e.g.* [28]). We outline a way to apply 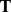 to these questions.

We close by reviewing open questions for 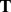, notably generalization, theoretical constraints and applications, and computational considerations.

## Analysis and Results


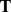
 is a stochastic matrix, created by superpositioning the deterministic dynamics of the networks supporting a set of input transitions. We interpret 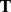 in three basic ways: (1) as a traditional Markov transition matrix, where the represented physical system has stochastic interactions, (2) as an uncertainty matrix, for the case where the system is deterministic but not (yet) uniquely determined, and (3) as a statistical aggregation, where the “system” is a population of deterministic individual systems with some shared and some varying behavior.

For (1), we are not aware of a physical system that switches between networks stochastically, but that idea shares some parallels to models of protein folding, specifically stochasticity in intermediate conformations leading to well-defined outcomes [29]. Nonetheless, we show its effectiveness as a model by demonstrating that 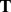 reliably approximates the distribution of point attractors (covered in **Attractors**). In a similar vein, we show how to apply traditional Derrida plots to 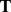 and propose that this may provide a way to characterize functions (covered in **Derrida Plots**), though our investigation into this measure is just beginning.

For (2), we calculated the Shannon entropy from 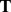 for different initial condition experiments. For those simulated experiments, we found the 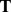-based method superior to the alternatives (covered in **Experiment Selection**). We also considered a scalarization of 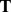 – the average and variability of Shannon entropies over each row – as a measure for comparing the uncertainty between different input dynamics; we did not find a compelling correlation between those measures and the number of experiments needed to specify the underlying network uniquely.

For (3), we outline how 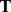 could make predictions about population-level response to experimentally induced environmental changes (covered in **Diversity Prediction**). We also show how a 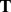 that accurately represents that population diversity could be used for relative risk and odds ratio calculations (covered in **Relative Risk & Odds Ratio**).

### Review of Boolean Networks & Formal Definition of T

#### Boolean Network Model and the Strong Inhibition Rule

The Boolean Network Model is

• a system of parts 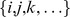,
• at time 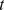, part 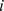 has Boolean state *active* (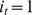) or *inactive* (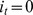), and
• an *update rule* gives 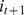 from system state at time 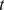 and interactions from other parts to 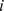: 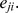

For our case, we use two types of interactions: *activating* (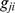) or *inhibiting* (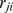) from part 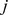 to 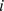. We also treated these interactions as Boolean variables: *e.g.*, 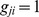 means 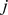 activates 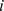, 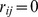 means 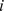 does not inhibit 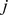.

We use a single rule to update all parts, typically referred to as *Strong Inhibition*:
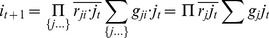
(1)expressed in Boolean algebraic operations: negation, 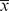, and extensions of AND (‘

’ to ‘
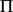
’) and OR (‘
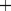
’ to ‘
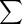
’) to set functions on a whole system state 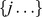. We show the rule with typical notation simplifications in the far righthand side.

The *Strong* qualifier emphasizes the effect of any active inhibiting interaction:
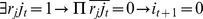



*i.e.*, any active inhibitor results in an inactive state, regardless of other signals. This formulation differs from previous published works, but that difference is not relevant to results; we explain why and our reasons for using this alternative definition in the **Supplemental Analysis: Strong Inhibition.**

A final note on the update rule and interactions: many boolean network models do not use a system-wide update rule paired with interactions, instead encoding individual rules for each part without reference to interactions. Of course, an overall rule plus varying interactions encodes “different” rules for each part; in some sense, the system-wide rule is a reductionist explanation of the individual rules. A single system-wide rule may prove too optimistic a reduction for many interesting systems; fortunately, 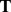 accommodates rules other than Strong Inhibition, as well as mixes of rules.

#### Boolean Dynamics

The Strong Inhibition rule is deterministic for 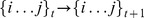. We call these single time-step state changes *transitions*, and the set of all transitions a system exhibits its *dynamics*. These transitions are uniquely identified by their before-and-after sets of active states, *e.g.* the system state 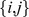 active goes to the state 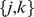 active. Hereafter, we abbreviate these sets with labels 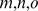, with transitions then written as 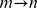 or just 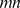.

As typical inputs, we have *partial* dynamics - not single transitions or complete behavior, but some subset of the total behavior. In particular, we analyzed time series terminating in an attracted state, *i.e.*:
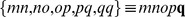



We denote collections like 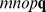 as a partial dynamic 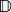. These time-series type dynamics cover many practical functions of interest: a starting condition triggering a cascade of known transformations ultimately returning to a stable state. We could also analyze a collection of the steady states, another highly practical application, or even a random assortment of transitions, but we do not have suitable source data for those cases.

For a Boolean system with 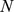 parts, there are 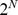 total transitions (one outgoing for each system state), and we are analyzing 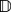
's with 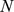 transitions. This is 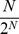 of the dynamics, or for the various system sizes we analyzed: about a half percent for the prototypical yeast system (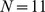), 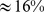 for the smallest systems (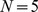), and less than a tenth of a percent for the largest systems (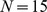).

#### The Measure T

We define the inverse of a dynamic, 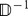, to be the set of all networks that exhibit 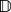 given the update rule(s). For the Strong Inhibition update rule, we used the algorithm in [1] to calculate the inverse (with simplifications from eq. (1)). However, the definitions below generalize to inversion results for other update rules, state and interaction types, *etc*.


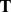
 is defined as the application of the set counting function 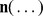 to 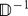:
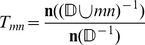
(2)


or, 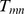 *is the ratio of the network class size for the observed dynamics with an additional constraining transition to the class size with no perturbation.* Put another way, 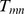 is the probability that, given a network chosen at random from the class 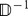, that network will also exhibit the transition 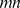. This equation is conceptually simple enough, but eq. (2) poses computational challenges as written; we discuss these in the **Supplemental Analysis: Computing** 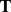.

For our results, we use an exact set counting function 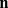 despite the computational challenges. However, we imagine that 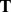 could be computed with sufficient accuracy, for certain applications, via an approximate 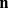 function and thus open larger systems up to analysis via this technique.

Finally, we must note: while 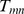 is the probability a particular network exhibits 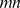, 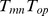 is *not* the probability that network exhibits 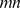 and 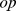, because rows in 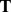 are *not* independent.

**Source Data:** As mentioned in **Boolean Dynamics**, we are working from a source data set of dynamic time series (terminating in an attracted state) for all of our results. These data cover network sizes 5, 7, 9, 11, and 15; for size 11 and smaller, we have 500k partial dynamics of each network size, and for size 15 we have 100 k. For each size category, the time series have the same number of transitions as the network size – *i.e.*, they have time steps 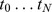. This data set comprises randomly generated dynamics targeting approximately 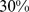 active states over the collected steps, filtered by those that have a solvable network under Strong Inhibition.

### Attractors

In Boolean dynamics, a point attractor is a system state which is static. 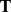
's rows correspond to states at time 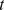 and its columns to states at 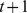, the diagonal entries 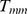 correspond to point attractor dynamics. Treating these 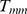 as Bernoulli trial probabilities (more on how to do so in **Supplemental Analysis: Attractors**), we can (1) calculate the distribution of point attractor counts (*i.e.*, the distribution of successful trials) and compare it to (2) the same distribution based on sampling the networks that support the dynamic generating 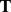 (more on sampling in **Supplemental Analysis: Sampling**).

## Results

**Figure 1:**
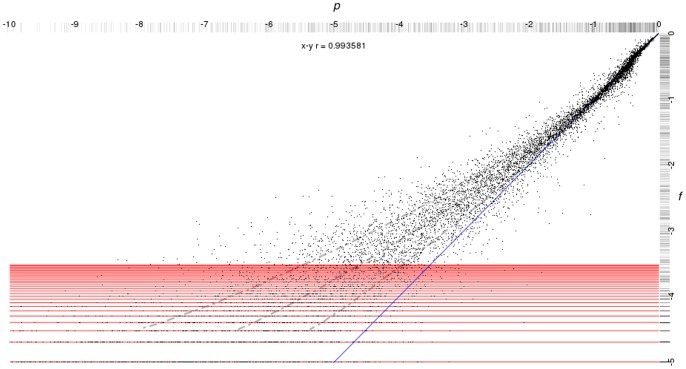
Point Attractor Distribution Correlations. This plot compares the distribution of point attractor number as calculated based on 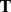 versus sampled. Each point, with base-10 logarithmic scales, is 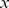, the calculated probability (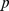) of some number (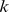) of point attractors based on 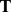 from a particular dynamic 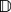, and 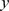, the sampled frequency (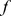) of that number of point attractors in networks that support 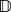; or, succinctly: 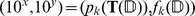. The plot shows that the 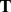-based distribution is tightly correlated with the sample down to about the 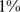 level, and then skews low. The plot is annotated with other information: both axes include rug plots to indicate point density, red horizontal lines indicating the 1 to 25 counts out the sample size (the region with visible notable “lines” of sample data), a blue 1-to-1 line for exact correlation, and dashed grey lines indicating the 20%, median, and 80% slices in the 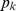 spread.

For each of the dynamics in our **Source Data**, we used 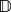 to compute 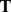 and provide a sample 10 k of the supporting networks. Individual network classes each yield different sample distributions of point attractors, but the resulting predictive performance of 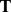 for those distributions is essentially identical. Figure 1 shows the computed versus sampled outcomes for the size 11 systems (the yeast cell cycle network system size), and is visually indistinguishable from figures generated for the other sizes. The resulting correlation coefficients across system sizes differ by less than 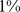 (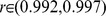), as do the resulting linear correlation parameters.

There are some caveats – as should be obvious from the figure (and to be expected, per **Supplemental Analysis: Calculating 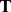
** discussion), the observed frequency departs from the calculated probability and the spread becomes relatively large for low probability events, even prior to entering the sampling size related noise region. That defect does not appear to be particularly relevant in practical scenarios. We found that almost all systems (1) have a few “high” (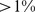) likelihood attractor counts followed by a variable tail, (2) this “high” region is most dense over all systems (the rug plots integrated into Figure 1 show this), and (3) the correlation in this region is higher and with linear slope 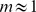 – *i.e.* perfect prediction. When we combined the distribution size categories for 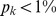 into 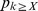 prediction, the departure from near-perfect prediction essentially vanishes. This might be a useful rule-of-thumb when applying 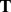, but we do not have an analytical argument for it, so extending that conclusion beyond the sizes we considered requires additional consideration. Finally, for the smallest systems (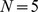), some of the systems had particularly entangled attractors – cases where pairs of attractors always occurred together or precluded each other; these cases could be practically addressed by computing the conditional probabilities for the excess attractors, given the low number of candidates for this size system. We may address this particular case as part of our more general take on higher-perturbation 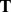
's.

For stochastic systems, these distributions of attractor sizes would be analytically exact. Thus, the 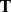-based distribution could be compared to experimental data from such systems to identify whether the analytic model is sufficiently constrained, includes the correct components, *etc*. However, we are not currently familiar with specific phenomena where such a comparison would be useful.

We conclude overall that 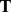 is suitable for estimating the point attractor count distribution for the network classes in our **Source Data** governed by Strong Inhibition. We suspect that more general network class criteria would also be suitably represented, especially when the application can tolerate lower resolution for distribution values at higher attractor counts. For detailed statistical applications, some additional work - *e.g.* confidence intervals on the distribution - would be required.

### Derrida Plots of T


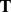
 can be used to measure functional stability, by making a graph akin to a Derrida Plot (recently applied in several Kauffman *et al.* publications on canalyzing interaction rules, and originally outlined in [30]). We demonstrate this by superimposing Derrida plots for the putative yeast cell cycle network and for 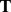 from the yeast cell cycle process. A Derrida Plot graphs Hamming distance between a sample of initial system states 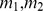 versus that of their subsequent states 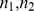, or 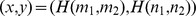. Also of interest is the Derrida coefficient, 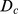, which is the slope of the plotted curve at the origin, and how it compares to 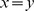.

#### Computation using T

Since a Derrida Plot conventionally measures deterministic Hamming distance, we need to develop a stochastic alternative. We propose that the classic expected value calculation of Hamming distance is a suitable stochastic substitute:
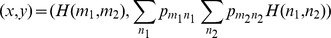



We define the matrix 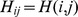, and after some manipulation based on matrix algebra obtain the much more calculable
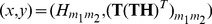
(3)over which it is straightforward to consider all points for a given system. When comparing to a particular network, eq. (3) works with a 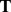 with the appropriate 1 and 0 values corresponding to the deterministic transitions.

#### Results

**Figure 2:**
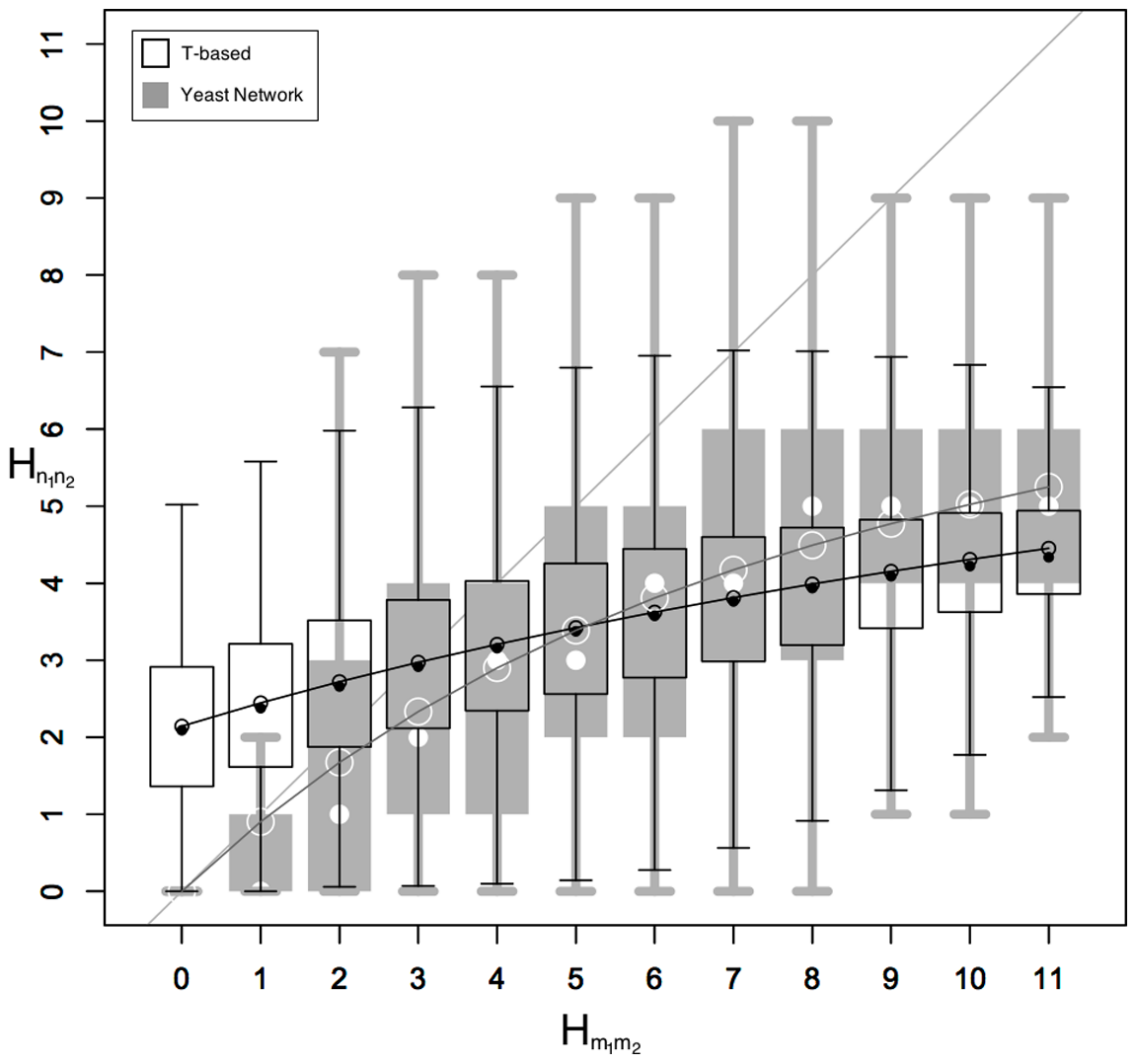
Derrida Plot Comparing Yeast Cell Network and Cell Cycle Process T. This plot compares the putative yeast cell cycle network (grey) and 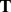 supporting the yeast cell cycle process (black). We have extended the plot to show median (solid points) plus box-and-whiskers in addition to means (open points). For this size system, we were able to evaluate a complete population instead of sampling. The putative cell cycle network is stable according to the Derrida coefficient: *i.e.*, the mean values form a trajectory below the 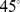 line. The 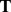-based results require more interpretation and context. Notably, states with 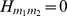 - *i.e.*, identical states - have 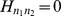 - *i.e.*, the same outcome - on a set network with deterministic rules. If we use 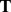 as a surrogate for such a network, then the resulting spread in 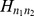 for 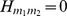 - essentially half the available range - should give pause when comparing the outcomes of nearby initial conditions. However, 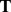 seems plausibly useful for estimating divergence for disparate initial conditions. On the other hand, if we believe our system is well represented by the superposition of networks, then that low 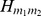 spread in 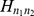 may provide insight into how (un)constrained the system noise is by the structured component.

For 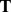, the outcome state is stochastic, so we calculated the expected value of 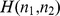 based on 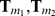. Figure 2 shows the combined plots, using box-and-whisker instead of the simple mean more typical of recent publications, and indicates several results. One, the putative yeast cell cycle network is ordered based on its 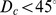. Two, for roughly a third to two thirds of the range, the 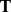-based results approximately represent the unique network results. For the middle third of the range, the boxes and midpoint-indicators (median and mean) are all overlapping, and for the latter third similar but less strong statements could be made. Finally, however: the 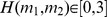 range poses some interesting questions for the 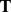-based curve. Notably, at 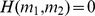 - where the initial conditions are identical -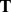 indicates a divergence of outcomes, which is impossible for a unique, deterministic network, but expected for a stochastic system. That presents an issue for the traditional 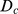 calculation, which is through the origin; it may, however, prove reasonable to simply offset and use the same slope criteria.

Obviously, this is a single point comparison. This result does not invalidate the idea of a function-based Derrida plot, but there is work to be done before considering it useful. We discuss that work in our concluding remarks, as well as proposing some preliminary interpretation of this single point result.

### Experiment Selection

When 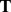 is generated from some partially observed dynamic, there remain many undetermined transitions and corresponding unknown interactions. Resolving those unknowns requires additional information, which must be garnered from either past or new experiments. However, there are typically many possible experiments to conduct and past results to search, and focusing on which would be most informative is a highly practical application of 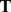. If we view the dynamics as partial information about a uniquely determined system, then using 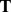 to calculate Shannon entropy for experimental selection is a natural approach.

#### Shannon Entropy Calculation

Each row 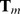 contains the transition probabilities for a particular initial condition, so the Shannon entropy for each row is
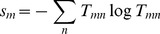
(4)where the particular logarithm base is only a scaling factor. This entropy indicates which initial condition we expect to yield the most information about the dynamics in an experiment observing 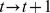 dynamics. As with the point attractor calculation, there are some caveats given that 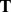 is a superposition. The rows are not independent, hence 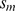 is only exact when ordering two initial conditions relatively, and since 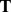 changes with each additional bit of information, the 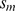 must be recalculated after an experiment.

However, our results indicate that such dependence may be irrelevant by comparing to a “naïve” entropy. This naive entropy is the uncertainty expected based on the Strong Inhibition rule and equiprobable interactions among parts (calculating this value is discussed in the **Supplemental Analysis: Naive Shannon Entropy**). This results in comparing how well we select experiments based on knowing something about the system dynamics versus only knowing the rules for interaction.

We also tested scalar measures that can be derived from 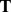:
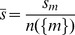
(5)which is the row-average Shannon entropy for the transitions; the variability and other higher order moments can also be derived as normal. We compared these values and a sample of the number of initial condition experiments to resolve a network across our source data and found no predictive power. This is retrospectively somewhat expected: this scalarization does not capture the (non-)independence of rows, which is in turn the principle indicator of whether resolving a particular row will also tend to the eliminate the uncertainty in other related rows. We posit that an alternative averaging procedure, one that weights by accounting for Hamming distances between the row initial conditions and perhaps some of the insights discussed in **Supplemental Analysis: Naive Shannon Entropy** section, might be more predictive.

#### Results

To compare experimental selection based on Shannon entropy, we simulated initial condition experiments on a network selected randomly from a class by calculating the transition from that initial state based on that particular network. We performed these simulations for 10 k network samples from each of the dynamics in our **Source Data.**

We selected initial conditions based on three orders: (1) Shannon entropy from 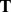, (2) Shannon entropy assuming only the Strong Inhibition rule and equiprobable interactions, and (3) at random. Each of these methods includes some form of updating after each experiment. For (1), we recalculated 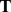 and identified the new most informative experiment; for (2) and (3), we excluded newly determined transitions (those not explicitly specified, but otherwise determined) from the possible experiment choices. Though we do not provide explicit results here, the distinction between (1) and (2) is qualitatively unchanged without the order updates, with both mainly just requiring more steps. The random method performs even more abysmally if determined transitions are not removed (often requiring nearly all states to be tested). Finally, for each ordering method, we resolve tied ranks by randomly selecting amongst the tied options.

**Figure 3:**
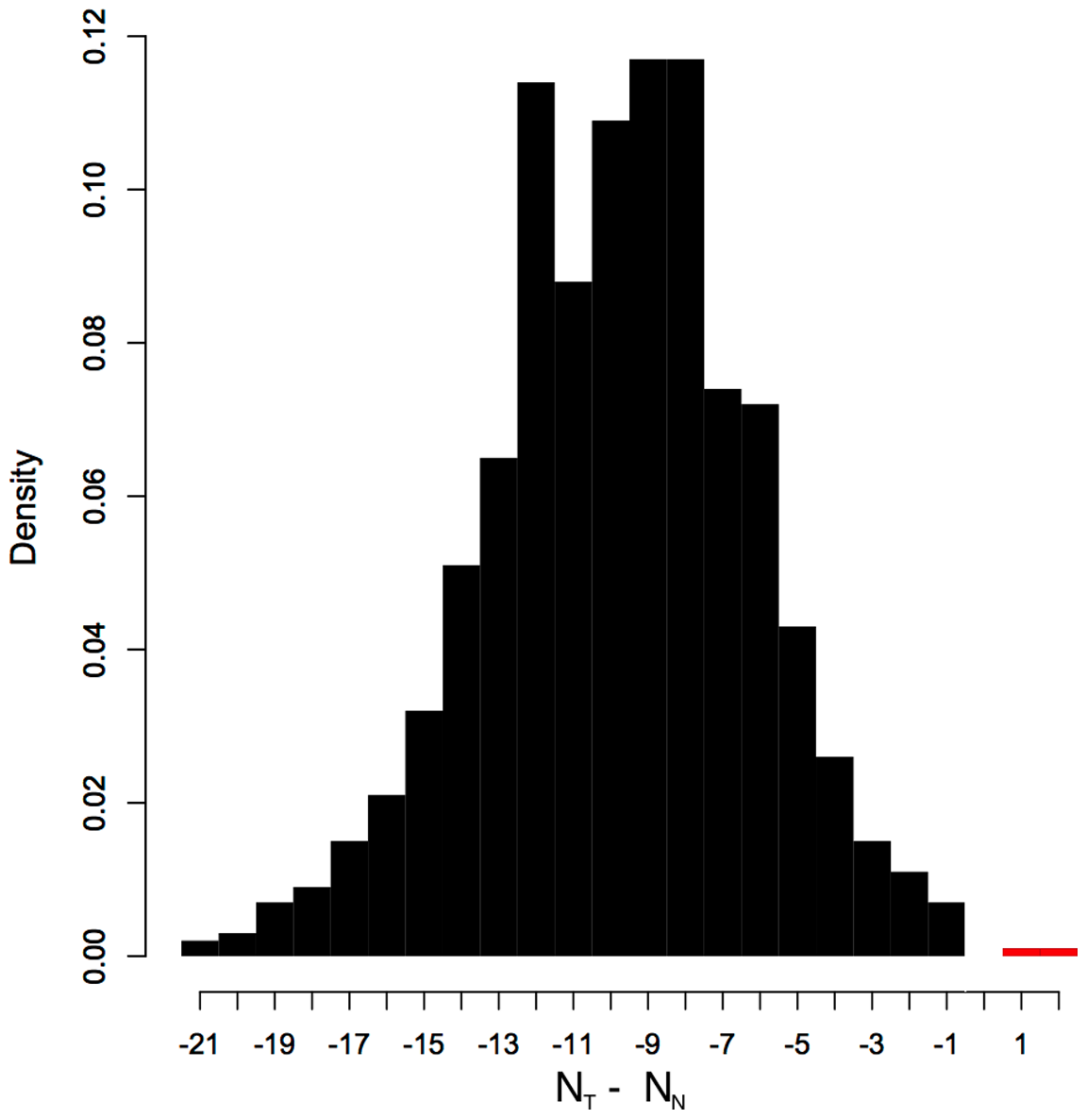
Experimental Selection. This plot is a histogram comparing number of experiments to determine an underlying network given partial initial information. The categories are the number of simulated experiments using 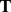-derived entropy (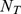) minus the number using using naive entropy (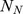), so the black region corresponds to the relative amount where 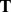 performs better, and the leftmost columns correspond to the greatest advantage. This sample mixes the results from considering 10 k supporting networks from each of our 11-element random dynamics. Similar results were obtained for other system sizes.

Figure 3 shows this comparison for all of our 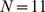 systems. We do not explicitly compare to selection at random here, because that method is so uncompetitive (typically requiring an order of magnitude more steps) that the plot perspective becomes uninformative. Using 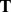-based entropy shows a substantive advantage over the naive entropy, with almost all of the sampled networks requiring fewer experiments and the typical networks requiring 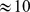 fewer experiments. Similar results appear for the other system sizes, with less advantage in smaller systems and more advantage in larger systems. We did not attempt to identify a scaling equation for this shift.

#### Receiver Operating Characteristic

As a complementary application to recommending which tests to perform, we also considered treating 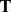 as the test itself by calculating the Receiver Operating Characteristic (ROC). ROC is a standard assessment of a test (early theoretical discussion in [31]), measuring the test's true positive rate (TPR) against its false positive rate (FPR) across acceptance thresholds.

It is not obvious how to determine ROC for 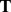 directly. Each 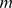 initial state can only go to one 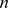 final state, but as the acceptance threshold varies, one arrives at the contradiction of having multiple results for a single 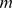. One plausible avenue might be to do sampling on 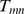 transformed across different “temperatures” (similar to a simulated annealing approach) and then using the mean curve (and perhaps the distribution about that mean as a further weighting refinement to the ROC calculation) to assess a particular 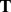. However, we think that sort of assessment warrants its own in-depth treatment.

**Figure 4:**
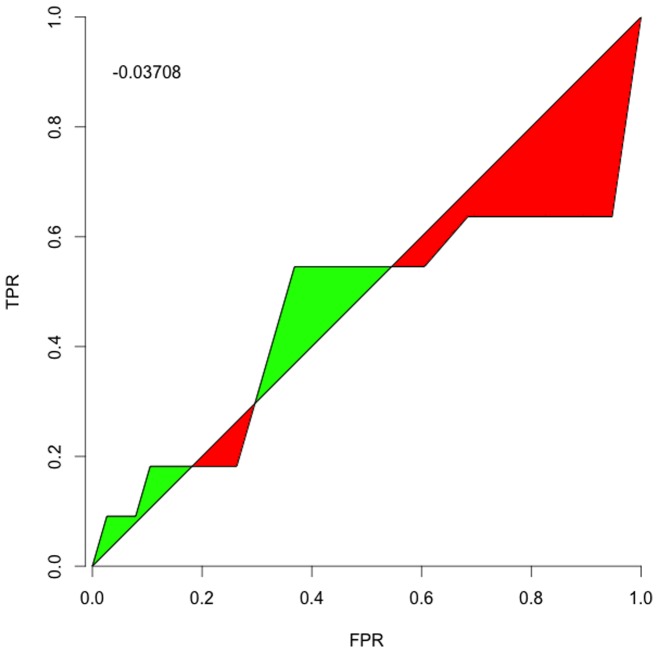
Inhibition Identification. The ROC for identifying the flexible and free inhibition interactions in the yeast cell network.

**Figure 5:**
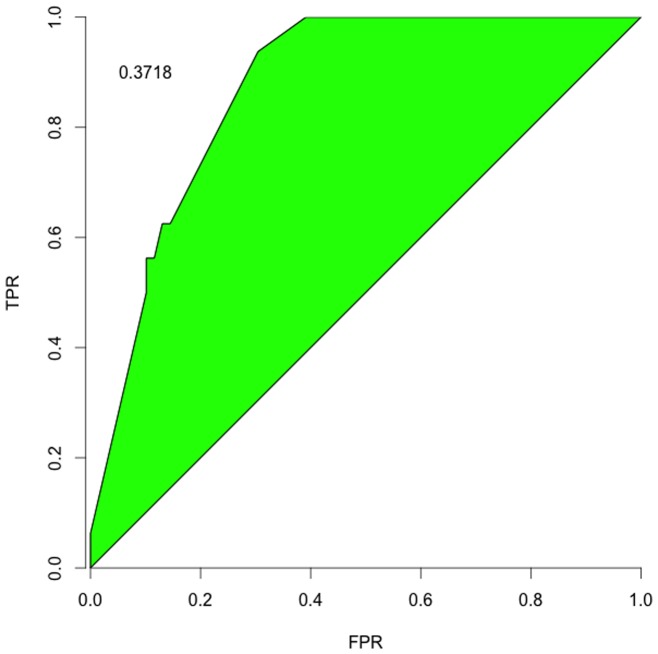
Activation Identification. The ROC for identifying the flexible and free activation interactions in the yeast cell network.

**Figure 6:**
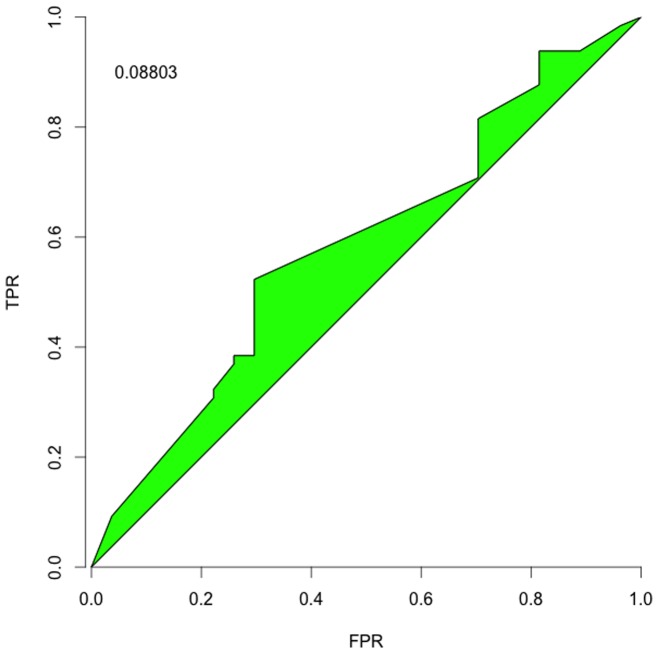
Non-Inhibition Identification. The ROC for identifying non-interactions between components in the yeast cell network.

So we instead opted to tackle a more straightforward question about the ROC for identifying interactions. Instead of using 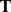, we created analogous matrices for the interactions
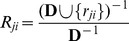
(6)

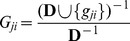
(7)

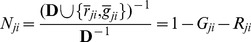
(8)and then assessed ROCs for each individual test; we did not attempt any of the advanced correlated-test ROC comparisons, again deferring that to a ROC-focused assessment of 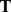 and 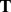-related measures. We set our TPR and FPRs based on reference to the putative yeast network, but excluded from those counts the cases where an exact outcome was known, *e.g.* 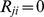 or 1. Excluding those creates a more conventional ROC curve, stretching from 0 TPR and FPR to 1 TPR and FPR, though perhaps including those points would be more informative. Figs. 4–6 show the curves and a typical summary statistic: *discrimination*, the area between the curve and 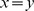. The discrimination values are 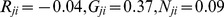 vs a maximum of 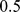; these would be higher (and all positive) if the curves included the exact outcomes.

### T as Population Aggregate

#### Diversity Prediction


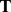
 may also indicate if a particular model is useful for representing population diversity. We borrow the notion of phenotypes from biology, defined explicitly here as meaning the categories of dynamic behavior that a network class will exhibit in response to initial conditions *not* part of the specification for that class. That is, every class network has an identical function, or phenotype, for the defining dynamics, but may exhibit diverse phenotypes to “off-nominal” or “noise” states.

To determine if a class-defining model does capture population diversity, we can make predictions based on that model and experimentally test them. In broad strokes, using a particular model – *i.e.*, number of parts, function 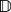, and optionally some fixed interactions – as input:

1. compute 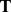,
2. conduct an experiment, translated to model states, that forces part(s) to be (in)active, precludes a fixed interaction, *etc*.
3. measure the population proportions of different responses
4. compute the proportion based on 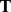 and the corresponding model (initial and outcome) states.

As a concrete example, posit that the putative yeast cell cycle process adequately models wild type yeast for the purposes of predicting their diversity. This could be tested by gathering or growing yeast under conditions that maintain diversity, then exposing them to an environmental change that affects the cell cycle and is included in the model. Continue to measure the growth rate of the yeast under this condition, and calculate the defect in that growth rate compared to typical conditions. From that, calculate the proportion of yeast suffering some inhibitory (or lethal) effect from that environment. Data obtained, identify which rows in 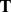 correspond to the environmental change (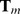), and some Boolean function 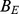 which converts outcome states (the 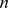 in 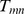) to normal growth (1) or abnormal growth (0). Then the experimentally affected fraction could be compared to the expected to be affected population fraction:
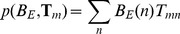
(9)


The researchers might discern order of magnitude effects (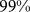, 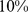, 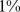, *etc* rough effect sizes), or at greater resolution depending on system, model, and experiment. If the effect orders agreed, they would have evidence supporting that the model represented sufficient constraints – instead of over or under constraining – to capture the system phenotypic diversity relative to that specific environment condition.

As to the question of a more general measure of phenotypic diversity surrounding a function, we have not yet set on a specific and useful calculation, but we suspect there may be a scalarization of 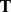 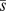 (as discussed in the **Experiment Selection** section) useful to that end, despite our initial failures in identifying one.

#### Relative Risk & Odds Ratios

A complementary application of 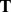 as a diversity model would to assessing relative risk or odds ratios of dynamic outcomes given additional conditions. Essentially, we take an event probability from 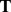
(*e.g.*, state 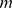 goes to any of 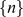 outcomes: 
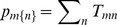
), and the same event probability from 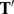, which includes the additional conditions in its calculation (*e.g.*, that some piece inhibits another, that some particular dynamic transition is always expressed). We then have the unconditioned and conditioned 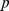
's, allowing simple calculation of
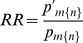
(10)

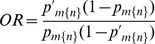
(11)These obviously enable traditional “population” comparisons for 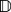 and 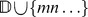, maintaining the caveat that rows in 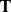 are not independent.

This framework also obviously allows comparison of 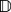
's with incompatible differences in some transition – *e.g.*, 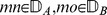. That is: given some, say, mutant yeast that exhibited marginally different cell cycle behavior, we could assess its relative likelihood of other dynamics that involved the cell-cycle components compared to the non-mutant strain.

## Discussion and Conclusions

We have shown that, for the Strongly Inhibited Boolean Network model, it is

• practical to compute the superposition stochastic matrix 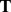 for small systems,
• accurate to use 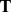 to calculate the point attractor distribution of systems supporting a particular dynamic, and
• useful to select experiments based on Shannon entropy from 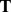

We have also shown how to calculate a Derrida plot and provide phenotype diversity predictions based on 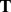. We consider 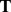 as an early step towards developing a more robust functionally oriented perspective to complement the largely structural current paradigm. We look forward to more work in this vein, and are excited about the prospective insights that will afford.

Our own investigations of open questions associated with 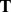 will include: (1) validating these results for other input dynamics, such as using attractor instead of time course data as input, and also incorporating some interaction constraints; (2) calculating 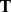 for other state and rule types, for example, generating 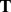 using different update rules across parts, or for ternary states instead of Boolean; (3) expanding the applications, for example considering basin size distributions and knockout experiments; (4) improving the computability of 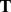 to addressing larger systems, by incorporating better exact and approximating algorithms from recent advances in the satisfaction counting problem (#SAT) [32]–[35]; (5) accounting for 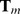 dependencies, possibly by identifying correcting two-state perturbation matrices, or by correcting the Shannon entropy from 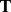 along the results for naive entropy.

We also proposed open questions specifically for the Derrida plots and phenotype diversity. Relative to the Derrida plot, there is an obvious general question about what exactly is being measured. Practically, we think comparing the plots across various functional inputs would provide a useful starting point. As part of that survey, we think that the small Hamming distance region deserves special attention; recall that, for the yeast cell cycle, this region presented a result that is impossible for a deterministic system. We posit that there may be a quantitative meaning to this result in light of the three interpretations we offered for 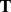: (1) that the divergence is real, because the system is stochastic; (2) that the divergence is an artifact, and indicator, of our uncertainty about the outcome; or (3) that the divergence is between different individuals in a population, not within a particular system. What different values for this divergence might ultimately mean, we do not know, but in light of those interpretations, we think it will be plausible to usefully compare across different input dynamics.

Relative to phenotypic diversity, interpretation of 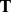 as a measure of supported functional diversity is plausible but needs work. One practical approach may be to compare 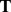
's for different functions in combination with comparing network samples in an evolutionary survival simulation. A summary entropy (*i.e.*, the sum of the individual row entropies) may also provide some insight about the accessible diversity.

## Analysis Supplement

### Strong Inhibition

Other publications invoking the Strong Inhibition rule present different formulations; eq. (1) is equivalent to those after adding interaction constraints dependent on the particular formulation. For example, most formulations do not allow self-inhibition: adding the 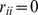 constraint recovers those models. Some formulations do not include decay: adding 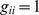 recovers those models.

Though these specific cases require an extra constraint, overall eq. (1) simplifies representation, reducing our algorithm's lines-of-code complexity without degrading performance and generalizing it to cover more phenomena within the same framework. This generalization comes from including self-directed interactions: if the part is already active, it can send a signal to stay active or deactivate. Allowing these self-interactions and having 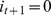 absent any signals, eliminates the need for special interactions to capture “self-degradation” or “decay”, used in several models including the putative yeast cell cycle, since these are naturally included in the expanded range. An especially pertinent point about the formal equivalence of eq. (1) to other published formulations, is that the conclusions developed about the inverse problem in [1] still apply because (1) there is an exact translation from (non-)decay interactions to self-activation interactions and (2) allowing self-inhibition does not fundamentally change any of claims in the steps of that proof.

The analysis sections are independent of this formulation (though the Shannon entropy calculation would require some trivial modifications), but we include it to limit any future confusion comparing our code base to this publication, and because we feel it is enough of an improvement to warrant general community adoption. Finally, this formulation is particularly conducive to representing variables–system states 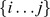 and interactions 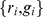
–by the 1-0 bits in an integer, which is the native underlying representation in most languages; we take advantage of this to use the typically faster bitwise integer operations in our inversion algorithm.

### Computing T

First, the number of transitions to be considered as perturbations grows rapidly with the system size 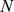: in a Boolean state system, there are roughly 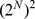 transitions, less the null state and small, constant number specified in 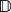. Second, counting the network classes resulting from the inversion procedure can be computationally expensive, so repeating that entire procedure for each perturbation becomes impractical.

However, we made the naive calculation of eq. (2) more practical with analytically equivalent modifications:

- Transitions can be calculated independently for each element, then combined into overall transitions. That is, for an initial *system* state (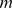), each *part's* state (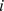) can be calculated independently, so we can consider the dynamics of a single part 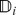 and perturbations to just that part's state. We define 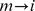 and 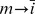 as 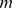 causes 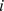 to be active or inactive, respectively, after a transition, and then:

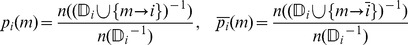
(12)Each 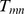 can then be calculated by multiplying the appropriate 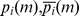 depending on which parts are active in 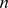.

- The definitions in eq. (12) are complementary, as we implied by their notation: 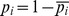. That is, an initial system state causes an individual part to transition to either 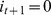 or 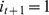, exclusively. Our code always calculates the latter, but there may be useful heuristics for identifying which is quicker to calculate.
- Finally, the additional transition constraints can be considered against the known results of 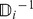. Modifying the Strong Inhibition inversion algorithm to add extra constraint clauses to an existing result is straightforward.

Taken together, these modifications substantially reduce the computation time. Anecdotally, on a two-core, 2.4 GHz system the yeast-sized systems (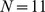) referenced in the introduction take order 1 day for the naive version of the calculation versus order 1 second with the above modifications. The independence of parts and the ability to easily introduce a new clause both contribute substantially, which indicates both are important practical considerations when calculating 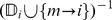 and thus 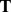 for other update rules.

#### Attractor Bernoulli Trials


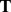
's diagonal represents the proportion of the class for which any particular state is a point attractor. We can use the diagonal elements as probabilities in a series of Bernoulli trials. We ignore known attractors (the null state, any specified in 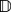, and any determined while calculating 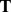) and the non-attractors (0 valued entires, also determined while calculating 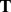) since these will be consistent across the class, and focus on the distribution of “excess” point attractors– *i.e.*, those that may or may not be present.

We calculated these distributions by repeatedly multiplying the previous distribution of attractor counts by 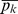 - *i.e.,* probability that 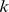 is not an attractor - and then adding that to the same distribution shifted over 1 and multiplied by 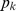 - *i.e.* probability that 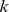 is an attractor. That is,
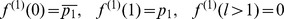





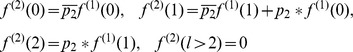


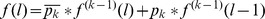


For cases where several of the 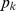 are equal (which typically happens when some set of the elements have the same behavior in the input dynamic), we could use binomial distribution results for faster and more accurate computation of those portions, then combine those in the same fashion described for the distinct Bernoulli trials (or via approximations as described in, *e.g.*, [36]). For the system sizes we considered, the frequency of this scenario did not seem to warrant the extra code, so we have not taken advantage of this possibility. It may be necessary for larger systems given the exponential expansion in possible point attractors.

### Sampling Networks

We obtained 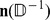 values large enough that enumerating all of the supporting networks, even for simple measures like point attractors and small system sizes, proved impractical. For the comparison of 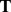-based results and statistical equivalents from the associated network class, it is necessary to sample that class since its size can exceed 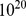. We generate the samples uniformly by using the free interactions and the enumerated interaction sets.

The free interactions are chosen uniformly from the available options: 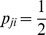 for activation (or inhibition) from 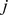 to 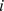 when inhibition (or activation) is forbidden (that is, 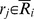 and 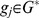 or vice versa), or, if neither type is precluded, 
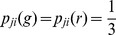
. For the enumerated component, we fix a list of the sets of satisfying interactions, and then randomly select (with replacement) items from the list. The fixed components - both required and forbidden - are consistent in all of the generated networks. This sampling procedure is consistent with previous published methods for uniform sampling [37].

We then calculate all of the pertinent dynamic transitions for each sample; *e.g.*, if the question is point attractors, then we examine only the possible point attractor states by excluding 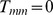 entries.

### Naive Shannon Entropy

In the Boolean network model using the Strong Inhibition rule, an initial state only has effects through its active parts. In the naive case, we have no knowledge about the interactions among these parts, so they are indistinguishable. A target part's probability of being active at 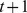 is then the probability of (1) at least one activating interaction from an active part at 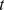 and (2) there being no inhibiting interactions from the active parts at 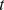; or, where 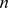 is the number of active elements in the precursor state:
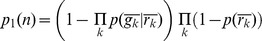



so given the equiprobability:
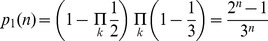


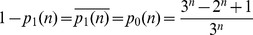



Of course, the entropy for a single element is
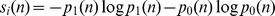
and since all of the elements are independent in their unconstrained behavior, the system entropy is simply scaled by the size of the whole system 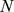. Since 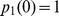 and 
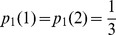
, and 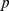 otherwise decreases with 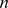, uncertainty decreases with larger 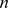. Thus, the best experiments, based only on the rules of Strong Inhibition, are 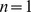 and 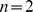. Though we did not use 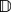 input with fixed interactions, that information could be incorporated into this calculation by replacing the known terms in product with either 1 or 0.
